# Genetic basis of phenotypic diversity in *C. stenophylla*: a stepping stone for climate-adapted coffee cultivar development

**DOI:** 10.3389/fgene.2025.1554029

**Published:** 2025-08-14

**Authors:** Paul M. Lahai, Peter O. Aikpokpodion, Alieu Mohamed Bah, Mohamed T. Lahai, Lyndel W. Meinhardt, Seunghyun Lim, Ezekiel Ahn, Dapeng Zhang, Sunchung Park

**Affiliations:** ^1^ Sierra Leone Agricultural Research Institute (SLARI), International Development Association (IDA), Kenema, Sierra Leone; ^2^ Department of Crops Science, Faculty of Agriculture, Njala University, Mokonde, Sierra Leone; ^3^ Department of Genetics and Biotechnology Faculty of Biological Sciences, University of Calabar, Calabar, Nigeria; ^4^ Department of Crop Science, Faculty of Agriculture, Eastern Technical University, Kenema, Sierra Leone; ^5^ Sustainable Perennial Crops Laboratory, United States Department of Agriculture, Agriculture Research Service, Beltsville, MD, United States

**Keywords:** Coffea stenophylla, genetic diversity, genome-wide association studies (GWAS), selective sweeps, adaptive evolution, genetic diversity, functional enrichment

## Abstract

Climate change poses significant challenges to global coffee production, particularly for Arabica coffee, which is constrained by a narrow temperature tolerance and a limited genetic pool. This study explores *Coffea stenophylla*, a species native to West Africa, as a potential alternative to Arabica due to its adaptability to higher temperatures and high-quality flavor profile. Using genome-wide association studies (GWAS), we investigated the genetic basis of phenotypic diversity within *C. stenophylla* accessions from Sierra Leone, focusing on traits related to growth habit, fruit and seed morphology, and plant structural characteristics. Our findings revealed significant SNP-trait associations that underscore the genetic diversity and potential of *C. stenophylla* for breeding programs. Additionally, we identified potential selective sweeps and conducted functional enrichment analysis, which highlighted genes involved in inflorescence development and flowering regulation, indicating adaptive evolution to local climates. These results suggest that *C. stenophylla* retains valuable genetic diversity that can be harnessed for developing improved cultivars better suited to the climatic challenges.

## 1 Introduction

Coffee, universally cherished for its rich flavor and stimulating properties, has become a crucial commodity in the global beverage market. The global coffee trade was valued at approximately $45.4 billion in trade in 2022 (ICO, 2022) and coffee cultivation significantly contributes to the economy of many developing countries, supporting the livelihoods of over 100 million coffee farmers (Vega et al., 2003; ICO, 2022). Notably, smallholder farmers, who own less than 5 ha of land, constitute approximately 95% of the world coffee farmers and are responsible for the majority of global coffee production, contributing an estimated 60%–80% (Siles et al., 2022; Poncet et al., 2024), which underscores the critical role of this commodity in global agriculture and socio-economic impact.

However, the sustainability of coffee production faces significant threats from climate change (Pham et al., 2019; Chemura et al., 2021). Challenges such as drought, heatwaves, and the overall rise in annual temperatures jeopardize the future of coffee production, presenting significant hurdles to maintain agricultural viability of coffee and ensure economic stability for communities reliant on coffee farming (Läderach et al., 2017; Davis et al., 2019). Despite the urgent need for sustainable agricultural practices to mitigate these impacts, progress remains slow (Adhikari et al., 2020; Cassamo et al., 2023), underscoring a critical gap in the global coffee industry’s response to environmental challenges.

While there are approximately 124 species in the *Coffea* genus (Hamon et al., 2017), the global coffee industry predominantly relies on just two species, Arabica (*C. arabica*) and Robusta (*C. canephora*). In 2022, global production was composed of about 56% Arabica and 43% Robusta (ICO, 2022). Arabica coffee is renowned for its rich flavor and aromatic superiority, but it is particularly vulnerable to the changing climate. This species thrives in a delicate temperature range, constraining its cultivation to a relatively narrow geographical belt that spans high-elevation areas in Central Africa and parts of South America (Bunn et al., 2015). The specificity of its growing conditions makes Arabica highly vulnerable to temperature variations, emphasizing the need for developing heat-tolerance varieties through plant breeding (Pham et al., 2019).

The effort to develop climate-resilient Arabica coffee is restricted due to the limited genetic diversity among Arabica coffee cultivars, which largely descend from a small pool of ancestral hybrids (i.e., Typica and Bourbon) (Scalabrin et al., 2020; Krishnan et al., 2021). This genetic bottleneck poses a significant challenge to breeding efforts to enhance resilience to climate change.

One interesting development in coffee breeding involves the utilization of the Timor hybrid (Clarindo and Carvalho, 2008), a natural crossbred between *C. canephora* and *C. arabica plants. This hybrid* exhibited strong resistance to coffee leaf rust, a devastating fungal disease, as well as tolerance to higher temperatures. Modern cultivars often incorporate traits from *C. canephora* through Timor hybrid-based breeding to enhance pathogen resistance and high-temperature tolerance (Romero et al., 2014). However, this approach has led to unintended consequences. While Timor hybrid-based breeding enhances resistance, it can also lead to lower-quality coffee (Bertrand et al., 2003). Additionally, backcrossing with Arabica to regain some of its flavor profile can result in a loss of the very disease resistance (Talhinhas et al., 2017). While other coffees, like Robusta and Liberica, can tolerate warmer, drier conditions than Arabica (Davis et al., 2006), none have been able to match Arabica’s taste profile, making them less commercially viable.

A historically neglected coffee species, *C. stenophylla*, has recently gained attention as a viable alternative to Arabica (Davis et al., 2020; Davis et al., 2021). This diploid species, capable of thriving at higher temperatures, has been recognized for its quality that rivals or even exceeds that of Arabica. Reintroducing this species into modern coffee cultivation could be a promising strategy for ensuring the crop’s resilience and future sustainability. This ancient coffee species suffered from decades of neglect, leading to the near extinction of its wild populations due to rampant deforestation and agricultural expansion (Davis et al., 2019). Despite its superior quality, the species traditionally exhibited lower yields compared to the robusta variety, making it commercially less attractive. To leverage this species for the benefit of the coffee industry, it is imperative to focus on breeding programs that aim to improve both its yield and quality. Such endeavors require a thorough understanding of the species’ genetic and phenotypic diversity as a foundation for successful breeding. Our previous investigation has shed light on the presence of genetically distinct regional populations within this species in Sierra Leone, revealing a reservoir of genetic diversity that had been presumed to be lost (Lahai et al., 2025). This finding highlighted the importance of conserving these wild populations, providing invaluable genetic resources for future breeding efforts.

The current study builds upon previous findings by employing regional populations of *C. stenophylla* for in-depth analysis. By aligning Genotyping-By-Sequencing (GBS) reads onto the *C. canephora* reference genome, we identified single nucleotide polymorphisms (SNPs) that are potentially crucial for protein functionality. These SNPs may play a pivotal role in defining the characteristic traits of the *C. stenophylla* species. Additionally, our GWAS analysis further elucidated significant SNP-trait associations, providing valuable insights into the genetic foundations of diverse traits among the coffee populations. In this analysis, we delved into potential candidate genes linked to trait-associated SNPs, establishing a framework for breeding strategies. By integrating genetic diversity, phenotypic characterization, and advanced genomic tools, our study contributed to the development of new coffee varieties with enhanced yield and resilience to heat and drought. These improvements could be achieved through the introgression of beneficial traits into Arabica coffee or by hybridization with high-yield Robusta coffee.

## 2 Materials and methods

### 2.1 Plant materials

A diverse panel of 143 *C. stenophylla* plants were sourced from their natural habitats (Ngegeru forest reserve, Kpumbu forest reserve, and Kasewe forest reserve) and the *ex-situ* field genebank of the Sierra Leone Agricultural Research Institute (SLARI) at Bambawo (Supplementary Table S1; Supplementary Figure S1). To identify selective sweep through comparative analysis, *C. canephora* (n = 24) and *C. liberica* (n = 23) plants were sampled from various regions including Kpumbu community, Njala community, Gbaimtanbandu community, and Pendembu coffee garden (Supplementary Table S2; Supplementary Figure S1). Leaf samples were collected following the protocol described by Lahai et al. (2025). During sampling, individual plant was subjected to a comprehensive phenotypic evaluation for eleven distinct traits, following qualitative assessment protocols (Lahai et al., 2023a).

### 2.2 Study sites and ecological characterization

*C. stenophylla* was collected from two key regions of Sierra Leone: Kenema and Moyamba districts, both recognized for harboring natural populations of this species (Lahai et al., 2023a). These areas are characterized by hilly terrains situated within forest reserve landscapes. In Kenema district, samples were collected from two primary locations: Kpumbu forest and Ngegeru forest. The Kpumbu forest lies at latitude 7°59′23.364″N and longitude −11°11′40.356″W, with an altitude of 375 m above sea level. The Ngegeru forest is located at latitude 7°56′50.634″N and longitude −11°12′16.818″W, with an altitude of 466 m above sea level. These sites are approximately 25 km and 10 km southwest of Kenema city, the headquarter of the Eastern region. Additional samples were obtained from the Kasewe hill forest reserve in Moyamba district, about 32 km northwest of Moyamba town in the Southern region. The Kasewe hill forest reserve is situated at latitude 8°19′11.694″N and longitude −12°10′1.62″W, with an altitude of 416 m above sea level. The mean annual rainfall is 2,546 mm for the Kpumbu/Ngegeru forest reserve and 2,135 mm for the Kasewe forest reserve, with an average monthly temperature ranging from 26 °C to 32 °C. The forests play a crucial role in biodiversity conservation, as the *C. steonphylla* populations exhibited substantial genetic diversity across these regions (Lahai et al., 2025). By selecting these study sites, the research aims to capture the ecological diversity and habitat characteristics necessary for understanding the distribution and adaptability of *C. stenophylla*.

### 2.3 SNP identification and imputation

Genomic DNA sequencing reads, obtained through the GBS method (Lahai et al., 2025), were aligned to the reference genome sequence of *C. canephora* (NCBI accession: GCA_900059795.1) (Denoeud et al., 2014) using bwa-mem2 (Vasimuddin et al., 2019). SNPs were initially filtered based on criteria including PHRED base quality ≥20, biallelic nature, support by at least 5 reads, and 90% of all aligned reads for homozygous genotypes, with a minimum of 2 reads supporting both reference and alternative alleles for heterozygous genotypes.

The initial SNP set underwent further refinement. Samples (n = 22) with >30% missing SNP genotypes were excluded, and SNPs with a missing rate of >20% and a minor allele frequency of <5% were excluded using the vcftools program (Danecek et al., 2011). Missing genotypes were imputed using Beagle v5.0 (Browning and Browning, 2016), which has demonstrated an average accuracy of >96.5% for illumina sequencing (Nothnagel et al., 2009). As a result, the imputation produced a variant call format (VCF) file comprising 12,706 SNPs across 121 *C. stenophylla* samples. SNP annotation (e.g., synonymous or nonsynonymous) and transition/transversion ratio analyses were conducted against *C. canephora* reference protein-coding sequences using SNPeff (Cingolani et al., 2012).

### 2.4 Phenotype analysis

The phenotypic data for 11 traits were categorized using a qualitative scale to capture the inherent variability of the plant characteristics under investigation based on at least 10 samples (Lahai et al., 2023a) (Supplementary Table S1). Growth Habit was evaluated as three scales (1 for open, 2 for intermediate, and 3 for compact); Stem Habit (1 for stiff and 2 for flexible); Angle of Primary Branches (1 for dropping, 2 for horizontal spreading, 3 for semi-erect); Young Leaf Tip Color (1 for greenish, 2 for green, 3 for brownish, 4 for reddish brown, and 5 for bronzy); Leaf Shape (1 for obovate, 2 for ovate, 3 for elliptic, and 4 for lanceolate); Leaf Apex Shape (1 for round, 2 for obtuse, 3 for acute, 4 for acuminate, 5 for apiculate, 6 for spatulate); Stipule Shape (1 for round, 2 for ovate, 3 for triangular, 4 for deltate, 5 for trapezium); Fruit Shape (1 for round, 2 for obovate, 3 for ovate, 4 for elliptic, 5 for oblong); Seed Shape (1 for round, 2 for obovate, 3 for ovate, 4 for elliptic, and 5 for oblong); Seed Uniformity (1 for uniform, 2 for mixed); Bean Size (1 for small, 2 for medium, and 3 for large).

Principal Component Analysis (PCA) was applied to the phenotype data to reduce dimensionality and identify underlying patterns explaining variance in the data. This analysis was performed using the ‘prcomp’ function in R statistical software (R core Team, 2024), specifying the center and scale parameters to ‘true’ to ensure that different phenotype data were mean-centered and standardized before analysis.

A biplot was generated to visually explore the relationship between phenotypes and principal components, using the ‘fviz_pca_biplot’ function from the factoextra R package v 1.0.7 (https://CRAN.R-project.org/package=factoextra). Variables (phenotypes) were represented as vectors, indicating how the original variables contribute to the principal components and how they relate to each other. The length and direction of the vectors were indicative of the contribution to the principal components and correlation among the variables. Hierarchical clustering was conducted using the “hclust” function in R statistical software (R core Team, 2024) based on pairwise Spearman correlation between the phenotype data.

### 2.5 Genome-wide association studies

Association analysis was conducted using the R-package, Genome Association and Prediction Integrated Tool (GAPIT) version 3 (Lipka et al., 2012) by employing the Settlement of MLM under Progressively Exclusive Relationship (SUPER) method (Wang et al., 2014). Population stratification was corrected by principal component analysis, with parameters adjusted for PCA total to 3 and model to SUPER. A Bonferroni correction threshold of −log10 [0.05/number of SNPs] set at 5.41 was used to identify significant SNPs associated with traits.

Subsequently, pairwise linkage disequilibrium (LD) was estimated between significant SNPs and nearby SNPs within a 100-kb window. LD blocks, defined by an *r* ^2^ value of at least 0.4, represent genomic regions where SNPs are co-inherited due to strong LD. If LD block was smaller than 100-kb, it was extended to 100-kb. Within these regions, candidate genes were identified using annotations from the *C. canephora* reference genome (Denoeud et al., 2014).

### 2.6 Functional annotation and gene ontology (GO) enrichment

Functional annotation and GO assignment for the candidate genes were conducted using the Trinotate pipeline (Bryant et al., 2017), as described by Park et al. (2021). Briefly, genomic total protein sequences were searched against the UniProtKB/Swiss-Prot database (non-redundant protein sequence database available at (https://www.uniprot.org) using diamond BLASTP program v 2.1.8 (Buchfink et al., 2021) and against the Pfam database (https://ftp.ebi.ac.uk/pub/databases/Pfam) with a cutoff of DNC using the hmmsearch function in the HMMER v3.3.2 (Finn et al., 2011). Based on sequence similarity, GO terms and biological functions from UniprotKB/Swiss-Prot database were assigned to the candidate proteins. GO term enrichment analysis was performed against the background of the total protein set using the phyper function in R statistical software (R core Team, 2024).

### 2.7 Positive selection analysis

To detect genomic regions under positive selection (selective sweeps) in the *C. stenophylla* population, we employed two methods: site-specific extended haplotype homozygosity (EHHS) and the ratio of EHHS between populations (Rsb), implemented in the R-package rehh (Gautier et al., 2017). EHHS values were first calculated for each SNP marker, and the values of *C. stenophylla* accessions were compared to a reference population consisting of two other species (*C. canephora* and *C. liberica*), collected from various regions of Sierra Leone (Supplementary Table S2). EHHS essentially reflects the likelihood that any two randomly chosen individuals from the *C. stenophylla* population share the same genetic makeup across a chromosomal region surrounding a focal SNP marker. Following EHHS estimation, the Rsb value was calculated for each SNP site. Rsb is a standardized ratio that compares EHHS values between the *C. stenophylla* and the reference population. Regions with an average Rsb exceeding 3 and P-value lower than 0.001 were identified within a sliding window of 1 megabase (Mb) with a step size of 100 kb. Overlapping regions were then merged to define putative selective sweep regions (Supplementary Table S3).

## 3 Results

### 3.1 Distribution and properties of SNPs in the *C. stenophylla* genome

For GBS-based genotyping, we selected 143 *C. stenophylla* accessions collected from four different regions in Sierra Leone (Supplementary Table S1). Following the imputation of missing genotypes and filtering SNPs for a minor allele frequency of 0.05, we obtained a total of 12,706 SNPs across 121 *C. stenophylla* accessions. The density of SNP markers per Mb varied, ranging from 1 SNPs on chromosome 3 to 93 SNPs on chromosome 5, with an average density of 34 SNPs across the genome (Supplementary Figure S2). Approximately half of the SNPs (n = 7,183) displayed a minor allele frequency of 25% or less, while 6,549 SNPs (52%) had an observed heterozygosity rate of 30% or less, calculated using a custom Perl script based on a formula: number of heterozygous individuals divided by the total number of individuals (Supplementary Figure S3; Supplementary Table S4).

**FIGURE 1:**
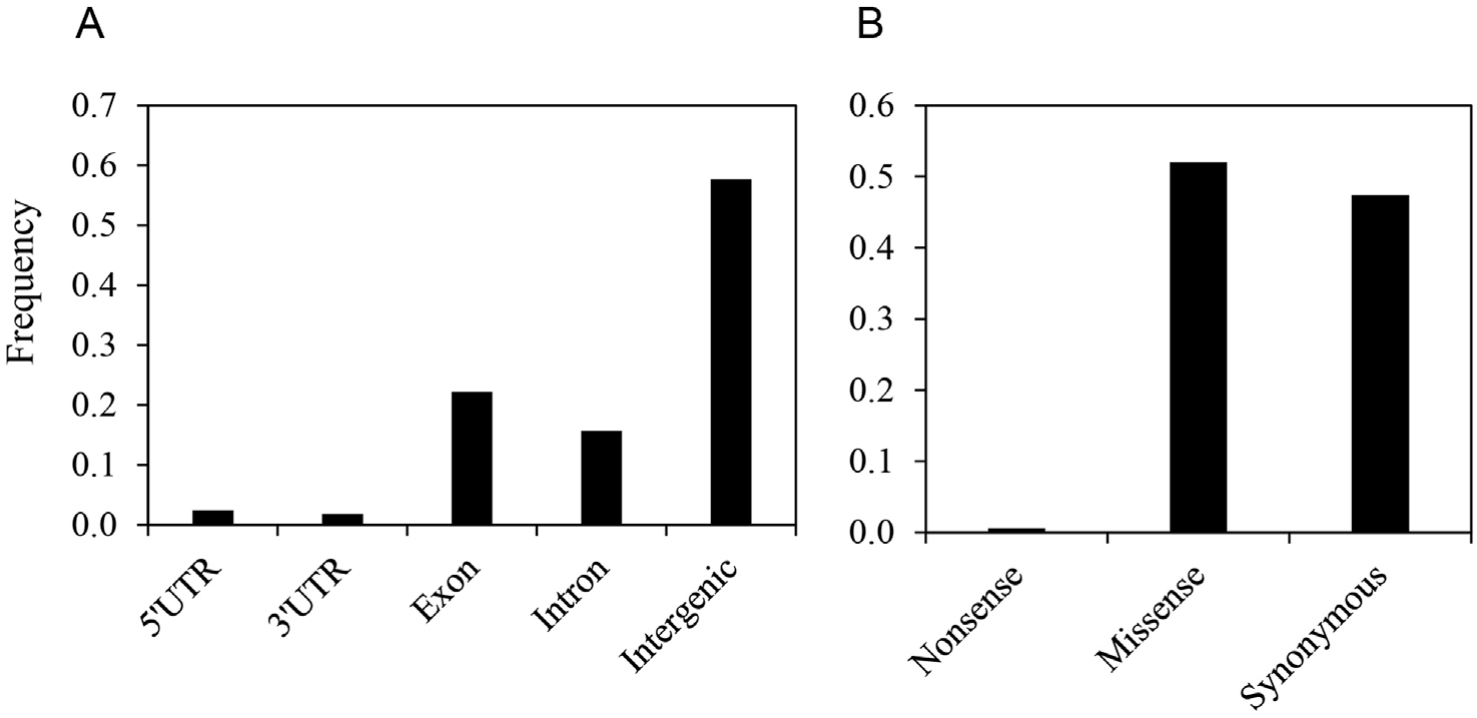
Distribution of single nucleotide polymorphisms (SNPs) in the genic region of the C. stenophylla genome. **(A)** Frequency of SNPs by regional location, categorized based on gene models from the C. canephora reference genome. **(B)** Frequency of synonymous and nonsynonymous SNPs within exon.

SNP loci were annotated based on predicted gene models from the *C. canephora* reference genome (Denoeud et al., 2014) to assess their impacts on protein sequences using SNPeff (Cingolani et al., 2012). Nearly half of the identified SNP loci (n = 7,336, 58%) resided within intergenic regions, while the remaining 42% (n = 5,370) were located within genes. Among genic SNPs, 37% (n = 1,998) were found in introns, and 52% (n = 2,822) were located within exons (Figure 1; Supplementary Table S5).

Exonic SNPs were further classified based on their predicted effect on amino acid sequences. Approximately half (n = 1,485) of the exonic SNPs were predicted to cause amino acid changes (i.e., nonsynonymous substitutions), including 17 SNPs that introduced premature stop codons. The remaining half (n = 1,337) were synonymous substitutions (Supplementary Table S5). Considering that all possible substitutions across codons are equally probable, a nonsynonymous substitution rate of 76% would be expected by chance (Wilke, 2004). Therefore, the observed lower rate of nonsynonymous substitutions suggested a strong selective pressure against such changes in the genes.

Furthermore, we analyzed the ratio of transition (substitutions between purine or pyrimidine bases) to transversion (substitutions between purine and pyrimidine bases) among all identified SNPs. Transitions (66.7%) were more prevalent than transversions (33.3%), resulting in a transition/transversion ratio of 2.0 (Figure 1). When restricted to exonic SNPs, the transition/transversion ratio was lower at 1.69, with transitions still dominating at 62.8% compared to 37.2% for transversions. This ratio is comparable to those reported in studies based on the kok-saghyz transcriptome (*Taraxacum kok-saghyz*) at 1.65 (Luo et al., 2017), rubber tree (*Hevea brasiliensis*) at 1.5 (Mantello et al., 2014), and chicory (*Cichorium intybus*) at 1.61 (Galla et al., 2016).

### 3.2 Functional enrichment analysis of genes affected by mutational impact in *C. stenophylla*

Among the SNPs identified in this study, specific variants were noted for their potential to disrupt protein function through translational or transcriptional modifications, such as premature stops, start or stop codon losses, and alterations in splice donor or acceptor sites. The SNPeff tool classified these SNP variants as “high impact”. In contrast, variants resulting in amino acid substitutions within genes were classified as “moderate impact”. To investigate the potential biological implications of these genetic modifications, we conducted a GO functional enrichment analysis on the affected genes. These genes were annotated with GO terms based on their homology to proteins cataloged in the UniProtKB/Swiss-Prot database.

The enrichment analysis of 32 genes (Supplementary Table S6) containing high-impact SNPs showed significant enrichment in functions associated with signaling and protein homeostasis, including “response to L-glutamate”, “positive regulation of receptor internalization”, “extraction of mislocalized protein from mitochondrial outer membrane”, and “cytoplasmic translational elongation”. Additionally, an intriguing enrichment was observed in a function associated with “viral genome replication” (Table 1), where mutations in these genes are likely to inhibit this function, potentially acting as a defense strategy against viral infections.

**TABLE 1:** GO biological processes enriched in genes associated with high-impact SNPs.

GO ID	GO Term	P-value
GO:0019079	viral genome replication	3.2E-03
GO:0002092	positive regulation of receptor internalization	6.4E-03
GO:1902065	response to L-glutamate	8.1E-03
GO:0140570	extraction of mislocalized protein from mitochondrial outer membrane	8.1E-03
GO:0002182	cytoplasmic translational elongation	8.1E-03

On the other hand, 978 genes with moderate impact SNPs were predominantly associated with stress- and defense-related processes. These processes are likely to play a role in plant adaptation and stress response. Significantly enriched pathways included “L-ascorbic acid biosynthetic process” (important for antioxidant stress) and “cytidine to uridine editing” (potentially affecting gene expression), followed by “negative regulation of ethylene-signaling” (stress) and “response to nematode” (defense) (Table 2). These findings highlighted the diverse functional pathways potentially influenced by genetic variations in *C. stenophylla*, requiring further investigation into their phenotypic impacts on the adaptation of *C. stenophylla*.

**TABLE 2:** GO biological processes enriched in genes associated with moderate-impact SNPs.

GO ID	GO Term	P-value
GO:0019853	L-ascorbic acid biosynthetic process	2.7E-03
GO:0016554	cytidine to uridine editing	5.0E-03
GO:0010105	negative regulation of ethylene-activated signaling pathway	9.4E-03
GO:0009624	response to nematode	9.5E-03

### 3.3 Phenotypic diversity among regional *C. stenophylla* populations

A previous study reported significant phenotypic diversity among natural *C. stenophylla* populations (Lahai et al., 2023a). In this study, we evaluated 11 phenotypic traits of *C. stenophylla* accessions: growth habit (GH), stem habit (SH), angle of primary branches on the main stem (APB), young leaf tip color (YTLC), leaf shape (LS), leaf apex shape (LAS), stipule shape (StS), fruit shape (FS), seed shape (SeS), seed uniformity (SU), and bean size (BS).

**FIGURE 2:**
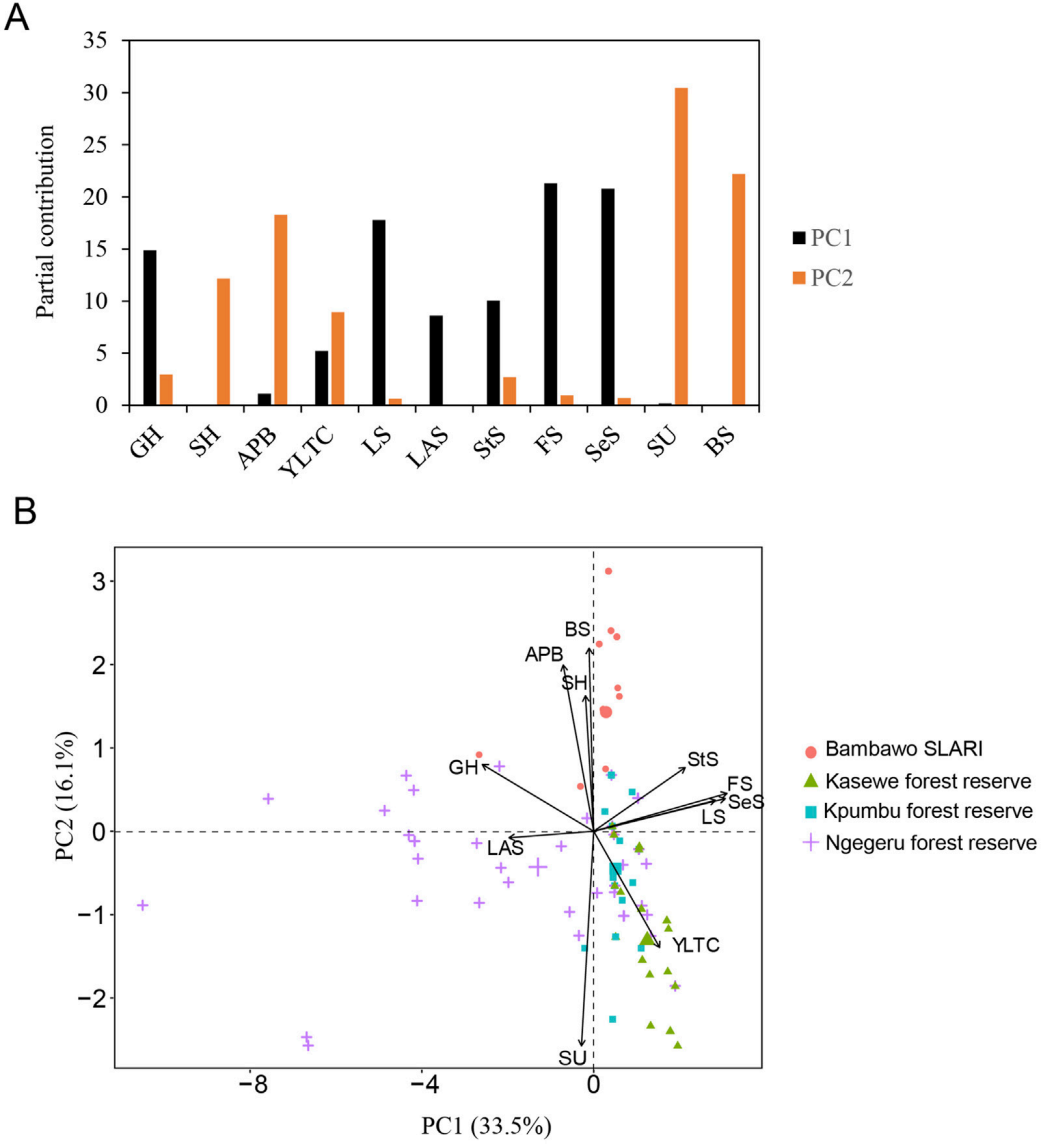
PCA biplot analysis of multivariate phenotypes in 143 C. stenophylla accessions. **(A)** Contribution of phenotypic variables to the top two principal components. **(B)** Biplot illustrating phenotypic similarities and the relationships between genotypes and phenotypic components. Different colors indicate the regional origins of the genotypes, while arrows represent the 11 phenotypic traits: LAS (leaf apex shape), GH (growth habit), APB (angle of primary branches), SH (stem habit), BS (bean size), StS (stipule shape), FS (fruit shape), SeS (seed shape), LS (leaf shape), YLTC (young leaf tip color), and SU (seed uniformity).

To elucidate the relationships between genotypes and phenotypes, we conducted a PCA biplot analysis with 121 accessions based on these 11 phenotypic traits. The first two principal components accounted for 50% of the total variation among the traits: the first principal component (PC1) explained 34% of the total variance and was primarily influenced by seven traits (GH, YTLC, LS, LAS, StS, FS, SeS), while the second principal component (PC2) accounted for 16% of the variance and was mostly influenced by five traits (SH, APB, YLTC, SU, BS) (Figure 2A).

The angle between vectors in the biplot indicated correlations among phenotypic components, with vectors pointing in similar directions being highly correlated. A high positive correlation was observed among APB, BS, and SH. These traits were then negatively correlated with SU and YTLC. Traits associated with morphological characteristics, such as FS, SeS, StS, and LS, displayed positive correlations, suggesting that the shapes of fruit, seed, stipule, and leaf are likely regulated by common genetic factors (Figure 2B).

**FIGURE 3:**
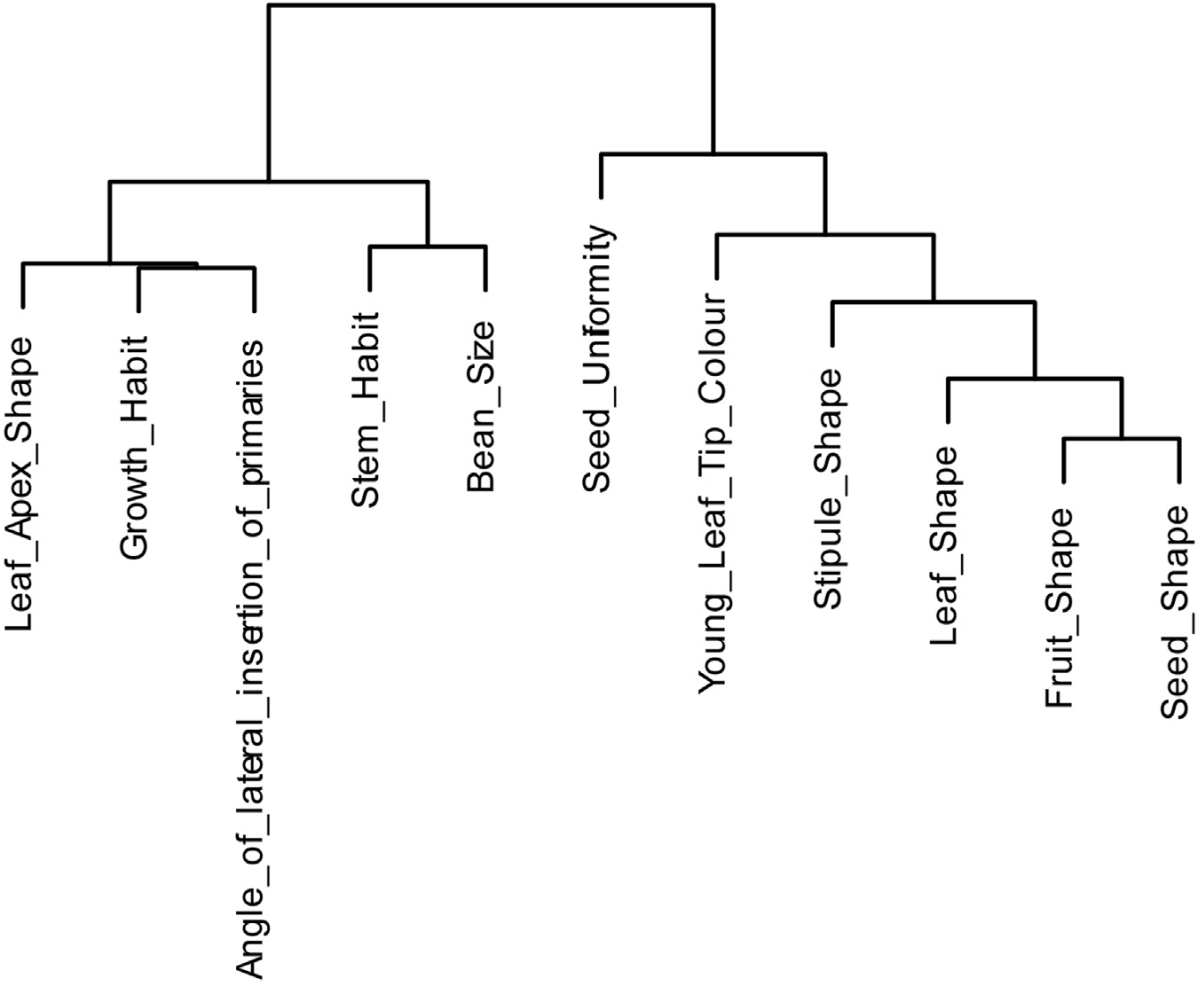
Hierarchical clustering dendrogram based on Spearman’s correlation among phenotypic traits. The clustering illustrates the relationships and grouping of phenotypes based on their correlation strengths.

Aligned with a biplot, clustering analysis based on Spearman’s correlation of the 11 phenotypes (Supplementary Table S7) revealed two distinct groups: one comprising LAS, GH, APB, SH, BS, and the other consisting of YTLC, SU and four morphological traits (StS, LS, FS, SeS). These two groups showed a weak negative correlation, as observed in the biplot (Figure 3).

Furthermore, the PCA biplot revealed three regional populations (Figure 2B). Bambawo and Ngegeru each formed a distinct group, while Kpumbu and Kasewe together formed another distinct group with an overlap between accessions from the two regions. The divergence of Ngegeru was primarily driven by PC1, contributed most by FS and SeS traits. In contrast, the separation between Bambawo and Kasewe/Kpumbu accessions was primarily driven by PC2, characterized mainly by SU, BS, and APB. Overall, these results indicated substantial phenotypic diversity among regional populations of *C. stenophylla* (Figure 3).

### 3.4 GWAS exploring the genetic basis of phenotypic diversity in *C. stenophylla* accessions

**FIGURE 4:**
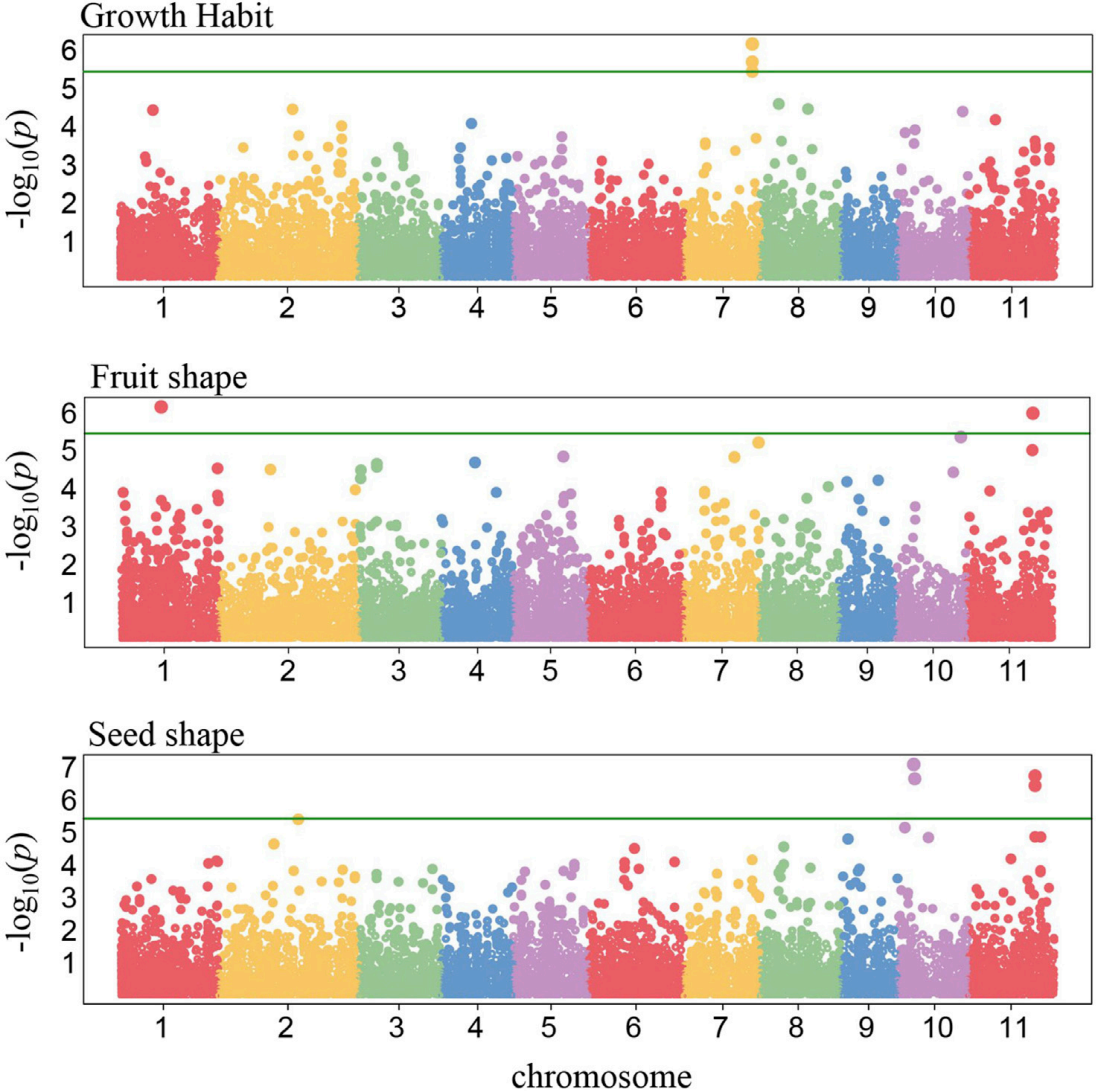
Genome-wide association signals. The x-axis represents chromosomal positions, and the y-axis represents −log_10_(*P*) values derived from mixed linear model association analysis. The green horizontal line indicates the genome-wide significance threshold after Bonferroni-correction (*P* = 3.9E-6).

**FIGURE 5:**
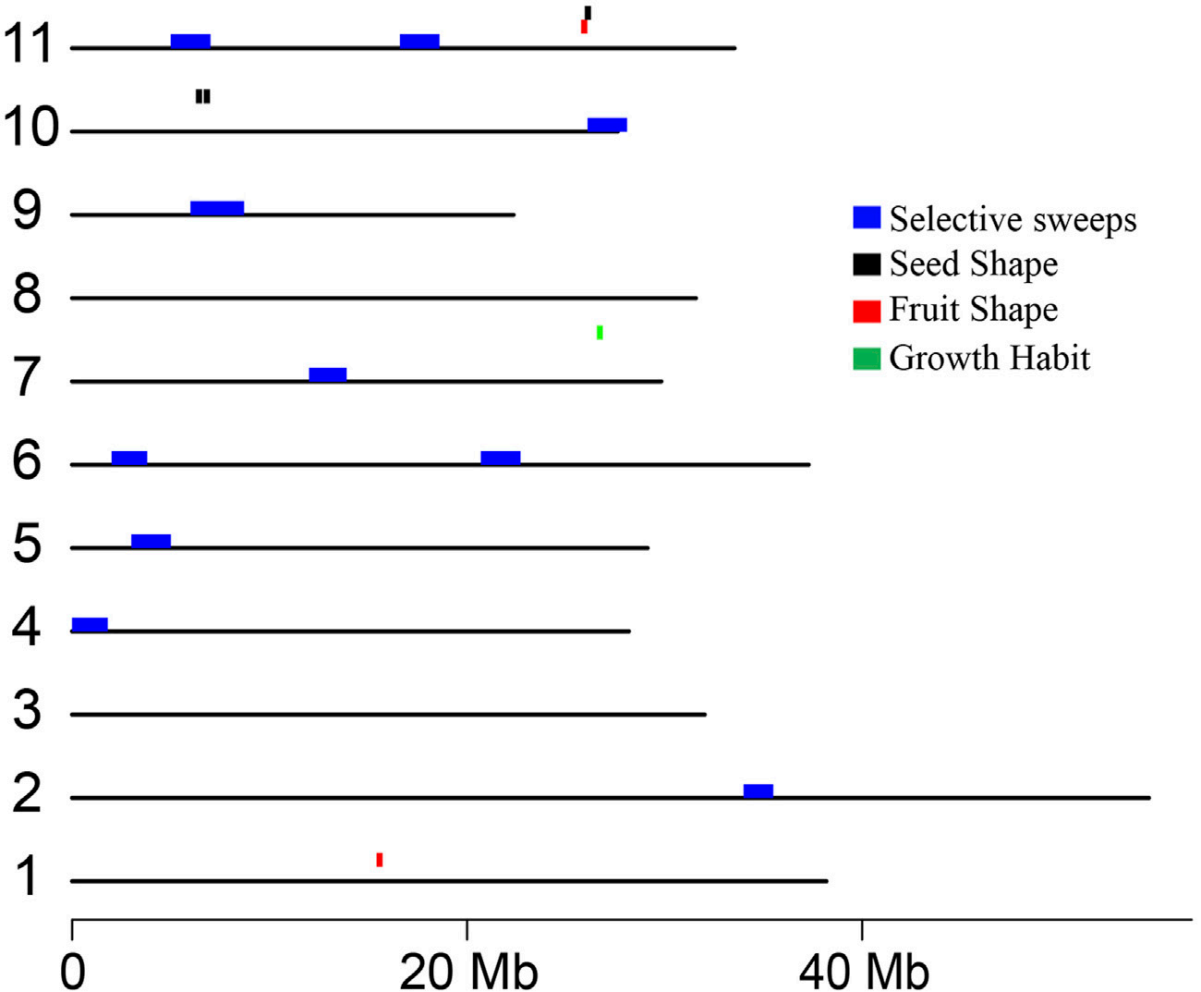
Distribution of potential selective sweeps in *C. stenophylla* and genomic regions identified through GWAS associated with three phenotypic traits. Selective sweep regions, characterized by low genetic variations, are highlighted in blue. Genomic regions associated with Seed Shape, Fruit Shape, and Growth Habit are represented in black, red, and green, respectively.

To identify the genomic regions associated with the 11 phenotypic traits, we conducted a GWAS analysis with 121 *C. stenophylla* accessions. Significant associations were identified for only three traits using a Bonferroni-corrected P-value threshold of 3.9E−6 (equivalent to −log10[P-value] of 5.4). Growth Habit and Fruit Shape each exhibited two SNPs significantly associated, while Seed Shape was linked to four SNP markers (Figure 4). The candidate genes for these three traits were selected within 100 kb LD blocks surrounding these significant markers. Nine candidate genes on chromosome 7 were associated with Growth Habit, while 16 candidate genes distributed on chromosome 1 and 11 were identified for Fruit Shape (Supplementary Table S8). Additionally, 26 genes on chromosome 2 and 11 were identified for Seed Shape (Supplementary Table S8). Consistent with the positive correlation observed between Fruit Shape and Seed Shape, associated genomic regions on chromosome 11 for both traits were in close proximity (approximately 183 kb apart) (Figures 4, 5). However, they displayed a moderate LD value of *r* ^2^ = 0.2.

Based on GO biological processes, functional enrichment analysis revealed significant enrichment for nine biological processes in genes linked to Growth Habit. The top three enriched functions included “protein O-linked mannosylation”, “dolichol metabolic process”, and “dolichol-linked oligosaccharide biosynthetic process”. These processes are associated with protein glycosylation and trafficking, potentially affecting protein function and localization (Supplementary Table S9). Other enriched processes included RNA processing, cellular organization, and developmental growth. For Seed Shape, candidate genes showed significant enrichment for functions associated with cellular trafficking, signaling pathways, and various aspects of plant growth and developmental processes. On the other hand, candidate genes for Fruit Shape were enriched for cell signaling involved in cell fate commitment and DNA metabolism. These findings underscored complex regulatory networks underlying these traits.

### 3.5 Identification of genomic regions under positive selection in *C. stenophylla*

Artificial selection (selective breeding) and natural selection, driven by factors such as local adaptation, bottlenecks, or environmental pressures, can leads to the rapid fixation of beneficial alleles, increasing their frequency within a population. This process reduces or eliminates genetic variation in regions surrounding the selected alleles, a phenomenon known as a selective sweep (Yamasaki et al., 2007; Turner-Hissong et al., 2020). In this study, we investigated whether selective forces have influenced genetic variation patterns in the *C. stenophylla* population. Our approach involved identifying genomic regions with relatively low genetic variation, suggestive of potential selective sweeps, by comparing the *C. stenophylla* genome with other species, such as *C. canephora* and *C. liberica*, using genome-wide SNP data. We detected ten potential selective sweeps in *C. stenophylla*, each spanning an average length of 1.9 Mb. Collectively, these regions accounted for 5.1% (18.6 Mb) of the genome and contained 1,154 genes (5.3%) (Figure 5). The genes within these selective sweep regions were enriched for functions related to inflorescence development (regulation of secondary shoot formation, maintenance of inflorescence meristem identity, positive regulation of flower development), transport process (glucose transmembrane transport, S-adenosyl-L-methionine transport, endoplasmic reticulum membrane fusion, exocytosis), and metabolic processes (histidyl-tRNA aminoacylation, L-alanine catabolic process by transamination, glyoxylate catabolic process, *de novo* IMP biosynthetic process) (Table 3; Supplementary Table S11).

**TABLE 3:** GO biological processes enriched in genes within the selective sweep genomic regions.

GO ID	GO Term	P-value
GO:2000032	regulation of secondary shoot formation	2.1E-05
GO:0010077	maintenance of inflorescence meristem identity	3.9E-05
GO:0006427	histidyl-tRNA aminoacylation	1.8E-04
GO:0016320	endoplasmic reticulum membrane fusion	1.6E-03
GO:1904659	glucose transmembrane transport	1.7E-03
GO:0006887	exocytosis	2.3E-03
GO:0009911	positive regulation of flower development	2.5E-03
GO:0019481	L-alanine catabolic process by transamination	3.2E-03
GO:0009436	glyoxylate catabolic process	3.2E-03
GO:0007064	mitotic sister chromatid cohesion	4.3E-03
GO:0015805	S-adenosyl-L-methionine transport	9.2E-03
GO:0045717	negative regulation of fatty acid biosynthetic process	9.2E-03
GO:0006189	“*de novo*” IMP biosynthetic process	9.9E-03

Furthermore, we examined whether the genomic regions associated with the three traits identified through GWAS analysis were involved in the selective sweeps. Interestingly, we found that none of the selective sweep regions overlapped with the genomic regions associated with the traits (Figure 5). This finding supported the notion that the genomic regions linked to these traits in *C. stenophylla* were likely not under recent selection pressures. Instead, these regions appeared to have been shaped by slower natural selection processes that may have occurred over multiple generations.

## 4 Discussion

Arabica coffee, constituting the majority of global coffee production, faces sustainability challenges exacerbated by climate change. As a cool subtropical crop, Arabica thrives within a narrow temperature range, restricting its cultivation to specific regions in South America and Africa. However, the development of new Arabica cultivars to address these challenges is constrained by a limited genetic pool, as most modern varieties originate from a limited number of ancestors (Ferreira et al., 2020; Zewdie et al., 2023; Scalabrin et al., 2024). In contrast, *C. stenophylla*, native to Sierra Leone and Ivory Coast, emerges as a promising alternative. This species flourish in low-elevation, high-temperature environments, and offer a high-quality flavor profile, making it a viable candidate for future coffee cultivation (Davis et al., 2020; Davis et al., 2021). Nonetheless, historical neglect and the risk of extinction in natural populations have limited its utilization.

A previous research (Lahai et al., 2023a) documented substantial phenotypic diversity within *C. stenophylla* populations, and further investigation (Lahai et al., 2025) demonstrated significant genetic diversity across regional populations. This current study corroborates these findings, illustrating that the observed phenotypic diversity in *C. stenophylla* is underpinned by genetic variation. Through GWAS, we identified significant SNP-trait associations, highlighting the Sierra Leone population of *C. stenophylla* as a valuable genetic resource for developing improved cultivars that address sustainability challenges in coffee production.

Among the 11 traits studied, the group, consisting of Angle of Primary Branches, Bean Size, and Stem Habit, showed a positive correlation among themselves, while exhibiting a strong negative correlation with Seed Uniformity (Figure 2B; Supplementary Table S7). This indicates that accessions with larger bean sizes tend to have more upright primary branches, as reflected by higher score for Angle of Primary Branches and Stem Habit (indicating flexible stems) rather than having dropping branches and stiff stems, while the accessions tend to have lower scores for Seed Uniformity (i.e., uniform seed size), indicating greater variation in seed shape and size. Despite these correlations, GWAS analysis could not find significant genetic associations for these traits, suggesting that their phenotypic variation may be primarily influenced by environmental factors rather than genetic determinants. For instance, favorable growth conditions could influence all three traits to move towards the uniform, larger bean sizes and more upright primary branches, consequently leading to the observed positive correlations among these traits. Interestingly, these traits are main contributors to PC2 variance in the PCA analysis, by which the Bambawo region is delineated from others. Plants in the Bambawo germplasm conservation site likely receive more consistent care compared to wild populations, which may explain the observed larger and more uniform bean sizes in this region. Further investigation into the role of favorable growth conditions on these traits would provide valuable insights.

Similarly, the group including four morphological traits (Stipule Shape, Fruit Shape, Seed Shape, and Leaf Shape) exhibited a strong positive correlation, while showing a negative correlation with Leaf Apex Shape and Growth Habit (Figure 2B; Supplementary Table S7). This suggests that the shapes of fruits, seeds, stipules, and leaves are likely regulated by common genetic factors, such that plants with round seeds are also likely to have round stipules, fruits, and leaves. Conversely, the negative relationship suggests that plants with an open growth habit tend to have narrow fruits, seeds, stipules, and leaves, while those with a compact growth habit are more likely to have round shapes for these organs.

Unlike the first group, GWAS analysis revealed significant genetic associations for Fruit Shape, Seed Shape, and Growth Habit, supporting the genetic regulation of these phenotypic traits (Figure 4). Furthermore, a strong positive correlation (Spearman’s correlation coefficient of 0.77) between Fruit Shape and Seed Shape aligns with GWAS findings, which identified closely located genomic regions associated with both traits on chromosome 11 (Figure 5). This proximity suggests a shared genetic control mechanism for these traits. However, their moderate LD value (*r* ^2^ = 0.2) also suggested that the SNPs could have independent effects on the traits, and the positive correlation may result from developmental or physiological linkages between the traits, rather than being controlled by the same genetic variant. Notably, candidate genes identified in these regions include those involved in cell division and cell fate, such as *CLAVATA3*-like genes, *WUSCHEL*-like genes, and Cell Number Regulator 7 and 9, suggesting an important role of cell division in the development of both fruit and seed shapes (Supplementary Table S6). Functional validation of these candidate genes is warranted to further unravel the mechanisms underlying fruit and seed shape determination in *C. stenophylla*.

Selective sweeps, characterized by the rapid fixation of beneficial alleles under strong selection pressures over a short period, can lead to a reduction in genetic diversity in adjacent genomic regions (Hartfield and Bataillon, 2020; Panigrahi et al., 2023). In this study, we identified genomic regions with significantly reduced genetic diversity, specifically within the *C. stenophylla* population compared to *C. canephora* and *C. liberica*. To understand the functional implications of these selective sweeps, we conducted a functional enrichment analysis of the genes in these regions and discovered a significant enrichment for genes associated with inflorescence development, such as “Maintenance of inflorescence meristem identity” and “Positive regulation of flower development” (Table 3). Given the critical role of flowering timing in adapting to local climates (Oh et al., 2008; Verhoeven et al., 2008; Ågren et al., 2017), this enrichment suggests that *C. stenophylla* populations have evolved under intense selective pressure to synchronize flowering with regional climate cycles. In Sierra Leone, the tropical climate is characterized by distinct wet and dry seasons (Lahai et al., 2023b): the 6-month wet season from May to October with heavy rainfall and high humidity, and the dry season from November to April. It is crucial for coffee plants to flower during the wet season to avoid adverse drought conditions of the dry season, ensuring successful reproduction and timely harvesting of coffee cherries from December through March (Lahai et al., 2023b).

Furthermore, we found a tandem array of paralogous MADS-box genes within these selective sweep regions, which are pivotal in regulating flowering time (Park et al., 2010; Ruelens et al., 2013). Clustered paralogous genes play a significant role in adaptive evolution through mechanisms such as coordinated regulation, subfunctionalization, and positive dosage effect (Koonin, 2005; Carretero-Paulet and Fares, 2012). For example, cold stress-related *CBF* genes, which exist in a cluster across many higher plants, enable plants to adapt to varying degrees of freezing conditions (Park et al., 2015; Park et al., 2020). Similarly, the MADS-box gene cluster in *C. stenophylla* may have evolved through gene duplication, and a positive dosage effect or subfunctionalization of these duplicated genes may benefit plants in fine-tuning the reproductive cycle to align with local climate conditions.

Beyond the MADS-box genes, we observed several other clusters of metabolism-related genes within the selective sweep regions (Supplementary Table S11). These included UDP-glycosyltransferase (n = 7), Cytochrome P450 (n = 11), Monothiol glutaredoxin-S2 (n = 7), Glucan endo-1,3-beta-glucosidase 8 (n = 4), Urease accessory protein D (n = 4), Wall-associated receptor kinase-like (n = 7), Probable caffeine synthase (n = 4), Tryptophan aminotransferase-related protein (n = 6), and Pleiotropic drug resistance protein (n = 7). These clusters suggest that the metabolic pathways may have evolved through natural adaptation to local environments, potentially contributing to the distinct flavor profile of *C. stenophylla*. Further research, including expression analyses and functional studies of these genes, would provide deeper insights into the adaptations and environmental pressures that have shaped this species.

## Data Availability

The datasets presented in this study can be found in online repositories. The names of the repository/repositories and accession number(s) can be found below: https://www.ncbi.nlm.nih.gov/, PRJNA1129264.
