# Supplementary material for: Genetic basis of phenotypic diversity in C. stenophylla: a stepping stone for climate-adapted coffee cultivar development
Source: Front Genet. 2025 Aug 14;16:1554029. doi: 10.3389/fgene.2025.1554029 (PMC12391046; doi:10.3389/fgene.2025.1554029)
Supplement: Supplementary file 1 [file Supplementaryfile1.docx]

**Genetic basis of phenotypic diversity in *C. stenophylla*: a stepping stone for climate-adapted coffee cultivar development**

Paul M. Lahai^1,2^, Peter O. Aikpokpodion^3^, Alieu Mohamed Bah^2^, Mohamed T. Lahai^4^, Lyndel W. Meinhardt^5^, Seunghyun Lim^5^, Ezekiel Ahn^5^, Dapeng Zhang^5^*, and Sunchung Park^5^*

^1^Sierra Leone Agricultural Research Institute (SLARI), IDA, Kenema 42215 and Sierra Leone

^2^Department of Crops Science, Faculty of Agriculture, Njala University, Mokonde 42215, Sierra Leone

^3^Department of Genetics and Biotechnology Faculty of Biological Sciences, University of Calabar, PMB 1115 Calabar, Cross River State 540271, Nigeria

^4^Department of Crop Science, Faculty of Agriculture, Eastern Technical University, Kenema 42215, Sierra Leone

^5^Sustainable Perennial Crops Laboratory, United States Department of Agriculture, Agriculture Research Service, Beltsville, MD, United States

***Correspondence:**

Dapeng Zhang ([dapeng.zhang@usda.gov](mailto:dapeng.zhang@usda.gov)) and Sunchung Park **(**[Sunchung.Park@usda.gov](file:///D:\SNP_sequencing_data\p4a_coffee_stenophylla_GWAS\stenophylla_GWAS_writing\Sunchung.Park@usda.gov))

# Supplementary Figures and Tables

## Supplementary Figures

**Figure S1.** Geographical distribution of sample collection sites of three coffee species in Sierra Leone (shaded grey region). The map illustrates the collection locations, with different colors representing regions and shapes indicating species: rectangles for *C. canephora,* circles for *C. liberica, and* triangles for *C. stenophylla*.

**Figure S2.** SNP density per megabase (Mb) across the genome with a 10 Mb sliding window. The x-axis represents the physical distance along each chromosome (Mb). The y-axis represents the chromosomal number, with a scale bar (0−50) at chromosome 1 representing the number of SNPs per Mb.

**Figure S3.** Distribution of SNPs based on (A) minor allele frequency and (B) heterozygosity. The distributions are presented as percentages of the total number of SNPs.

## Supplementary Tables

**Table S1.** List of *C. stenophylla* accessions with their place of origin and corresponding phenotypic scores

**Table S2.** Sampling sites for C. canephora and C. liberica plants

**Table S3.** Selective Sweeps identified using *Rehh* method

**Table S4.** Summary statistics for SNPs

**Table S5.** Functional annotation of SNPs based on *C. canephora* genome annotation, including their genomic location and associated amino acid change

**Table S6.** Functional descriptions of genes associated with high-impact SNPs

**Table S7.** Pairwise Spearman's correlation among 11 traits

**Table S8.** Candidate genes associated with agronomic traits identified through GWAS

**Table S9.** Gene Ontology functions enriched in genes associated with Fruit Shape, Growth Habit, and Seed Shape.

**Table S10.** Location of selective sweeps in the *C. stenophylla*

**Table S11.** Genes identified within selective sweep regions
